# Alien Marine Fishes Deplete Algal Biomass in the Eastern Mediterranean

**DOI:** 10.1371/journal.pone.0017356

**Published:** 2011-02-22

**Authors:** Enric Sala, Zafer Kizilkaya, Derya Yildirim, Enric Ballesteros

**Affiliations:** 1 Centre d'Estudis Avançats de Blanes, Consejo Superior de Investigaciones Científicas, Blanes, Spain; 2 National Geographic Society, Washington, D. C., United States of America; 3 SAD-EKOG, Maltepe, Ankara, Turkey; National Institute of Water & Atmospheric Research, New Zealand

## Abstract

One of the most degraded states of the Mediterranean rocky infralittoral ecosystem is a barren composed solely of bare rock and patches of crustose coralline algae. Barrens are typically created by the grazing action of large sea urchin populations. In 2008 we observed extensive areas almost devoid of erect algae, where sea urchins were rare, on the Mediterranean coast of Turkey. To determine the origin of those urchin-less ‘barrens’, we conducted a fish exclusion experiment. We found that, in the absence of fish grazing, a well-developed algal assemblage grew within three months. Underwater fish censuses and observations suggest that two alien herbivorous fish from the Red Sea (*Siganus luridus* and *S. rivulatus*) are responsible for the creation and maintenance of these benthic communities with extremely low biomass. The shift from well-developed native algal assemblages to ‘barrens’ implies a dramatic decline in biogenic habitat complexity, biodiversity and biomass. A targeted *Siganus* fishery could help restore the macroalgal beds of the rocky infralittoral on the Turkish coast.

## Introduction

**Figure 1:**
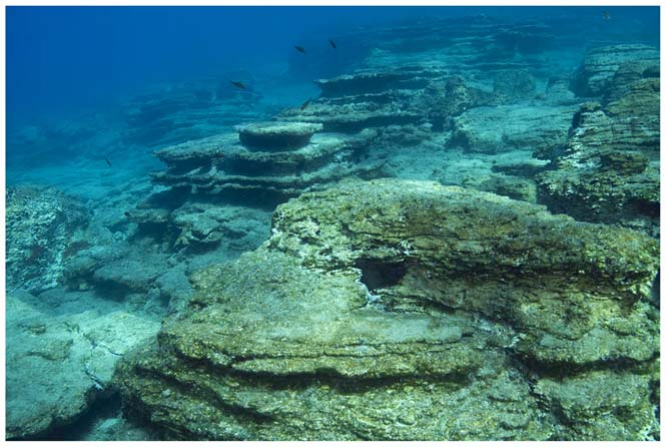
Infralittoral barrens at 10 m depth near Kas, Turkey, May 2008. Note the absence of erect algae and sea urchins.

The Mediterranean is a microcosm of the major threats to the oceans – historical overfishing, habitat degradation, pollution, introduced species, and global warming [1]. On shallow rocky habitats, the confluence of some of these stressors helps to create one of the most degraded underwater communities in the Mediterranean – infralittoral barrens. These barrens are impoverished bottoms dominated by bare rock with a few species of encrusting algae, caused typically by the grazing action of abundant sea urchins *Paracentrotus lividus* and *Arbacia lixula* [2], [3], [4]. In 2008 we observed extensive areas (several hundred meters in length) devoid of any erect macroalgae at several locations in the Mediterranean coast of Turkey, but sea urchin abundance was surprisingly low (Fig. 1). These areas (hereafter called ‘barrens’) were similar to sea urchin barrens but with small turfs of small filamentous algae and *Padina pavonica* during the season of highest algal production (spring-summer), although afterwards most visible algae disappeared (see results).

Plausible explanations for the existence of these barrens included low nutrient concentrations and fish grazing. We discarded nutrient concentrations as the cause because benthic algae were abundant on the uppermost wave-washed infralittoral zone, and also abundant in other locations situated only kilometers away. Herbivorous fish appeared to be abundant at the barrens, especially the alien rabbitfish *Siganus luridus* and *S. rivulatus*, which formed schools of up to several hundred individuals each.

*Siganus rivulatus* entered the Mediterranean through the Suez Canal in 1927 [5], and *S. luridus* appeared in 1956 [6]. Since their introduction they have established large populations in the Eastern Mediterranean [7], and *S. luridus* has even been found in the western Mediterranean as far as Marseille [8]. These alien fish, however, had not been reported to have a dramatic effect on the Mediterranean benthos [9].

**Figure 2:**
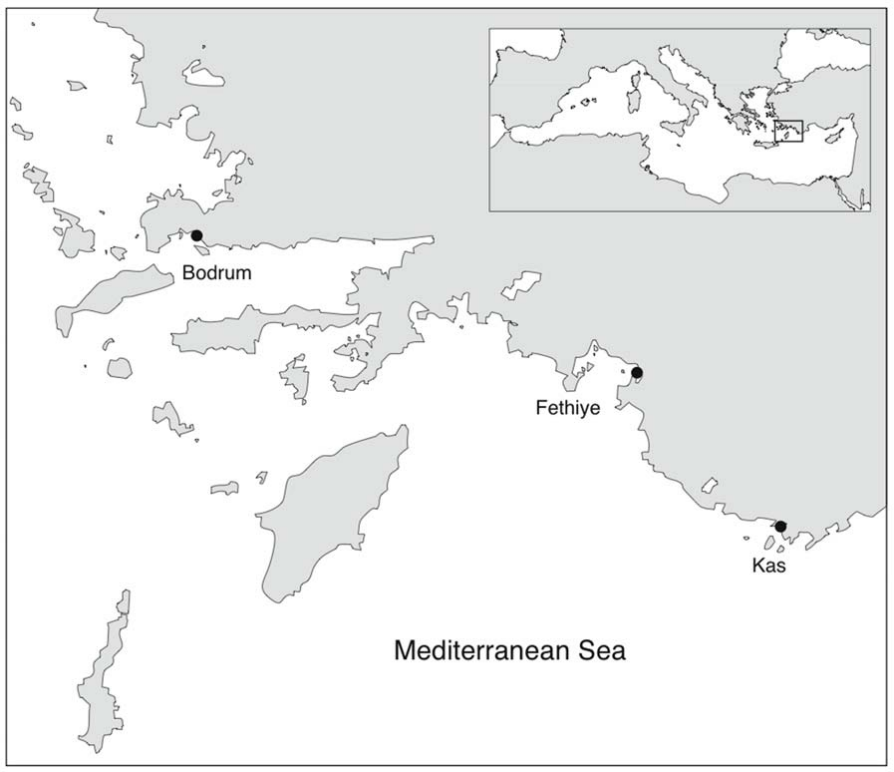
Location of study sites.

To test whether fish grazing – and in particular grazing by *Siganus* – was responsible for the creation of the observed denuded areas we conducted a fish exclusion experiment at three sites (Fig. 2) on the Mediterranean coast of Turkey.

## Results

The biomass of herbivorous fish represented between 46% and 57% of the total fish biomass at the study sites (Table 1), and it was significantly greater than the biomass of other fish trophic groups at Kas (ANOVA, p<0.001) and Bodrum (p<0.01). The rabbitfish *Siganus luridus* and *S. rivulatus* together accounted for between 83% and 95% of the biomass of herbivorous fish at the study sites (Table 2). *Sparisoma cretense* was the only other major herbivorous fish observed at the study sites, but its biomass accounted for between only 5% and 17% of total herbivore biomass. The major native Mediterranean herbivorous fish *Sarpa salpa*, and sea urchins were not observed within our transects at the study sites.

**Table 1:** Biomass of fish trophic groups (g m^−2^; mean ± S.E., n = 6) and percentage of total fish biomass at the three study sites.

Site	Apex predators	Carnivores	Herbivores	Planktivores	Total
Kas	0.05±0.05	2.38±0.84	6.17±0.80	4.17±0.95	12.77±3.78
%	0.4	32.6	48.3	18.6	
Fethiye	0.42±0.17	1.53±0.60	4.41±2.09	3.30±1.40	9.66±8.30
%	4.3	34.2	45.7	15.8	
Bodrum	0.15±0.15	2.07±0.95	7.03±1.20	4.02±0.89	13.27±6.52
%	1.2	32.6	57.1	16.8	

**Table 2:** Percentage of the total herbivorous fish biomass accounted for by the four major herbivorous species.

Site	*Siganus luridus*	*Siganus rivulatus*	*Sparisoma cretense*	*Sarpa salpa*
Kas	52	31	17	0
Fethiye	28	55	17	0
Bodrum	24	71	5	0

**Figure 3:**
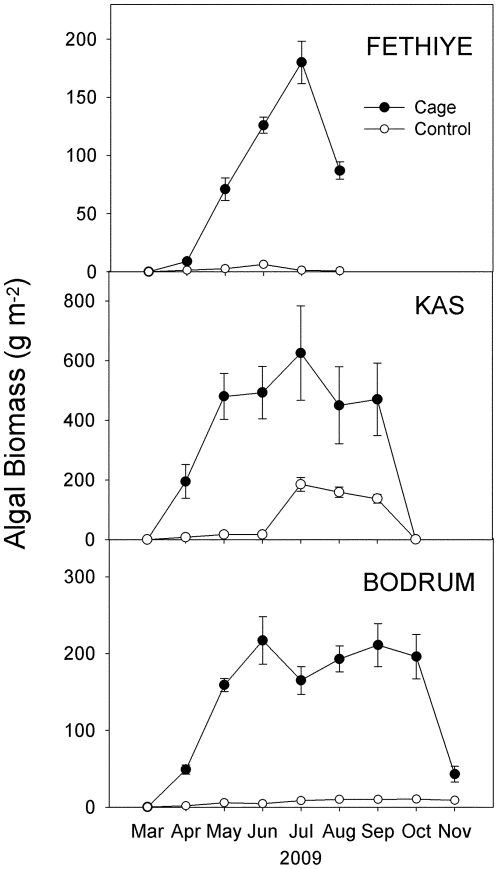
Evolution of total algal biomass inside the fish exclusion cages and controls (mean ± S.E., n  = 12) at the three study sites.

**Figure 4:**
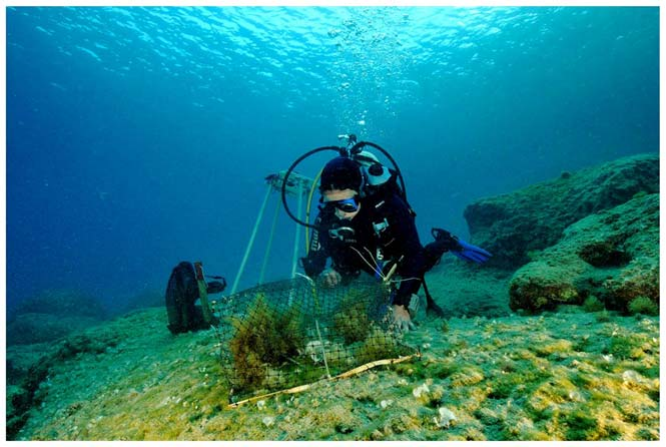
An exclusion cage at Kas in August 2009, five months after the beginning of the experiment. Inside the cage there is a well-developed algal assemblage, in contrast to the surroundings where only a few *Padina pavonica* stand out.

At the beginning of the experiment in March 2009, algal biomass was less than 5 g m^−2^ (Fig. 3). Algal biomass, including abundant canopy-forming brown algae of the genus *Cystoseira* (Fig. 4), grew significantly more inside the cages than in controls at all sites (ANOVA; interaction time × treatment, p<0.001; Fig. 3), but the increase in algal biomass in cages relative to controls was significantly different between sites (interaction site × time × treatment, p = 0.03). Algal biomass inside the cages at Kas was significantly greater than at Fethiye and Bodrum (p<0.001), but there were no significant differences between Fethiye and Bodrum. Algae declined in summer at Fethiye and Kas, and after October in Bodrum.

The best-developed algal assemblage inside the cages at Kas included a canopy of *Cystoseira* spp. (mostly *C. compressa*); an understory of *Dictyota* spp., *Hydroclathrus clathratus*, and *Padina pavonica*; and a turf of small filamentous algae. Although the benthic communities observed were not bare-rock-barrens like those caused by sea urchin grazing, they were largely devoid of erect algae, and colonized by a microscopic carpet of diatoms and turf of filamentous algae. The algal assemblage outside the cages at the peak of the production period at all sites was limited to small *Dictyota* and *Padina* turfs.

## Discussion

Our results show the most destructive effect of any alien marine fish in the Mediterranean marine ecosystem reported to date. The exclusion experiment clearly showed that without fish grazing, a well-developed algal assemblage would colonize the denuded habitats at the three study sites. We did not find sea urchins in our quadrats, and biomass of the two species of rabbitfish, *Siganus luridus* and *S. rivulatus*, was dominant. The massive recruitment of *Siganus* at the end of the experiment resulted in grazing inside the cages and the elimination of their algae within a month. The other herbivorous fish that may have an impact on the benthic algae is the parrotfish *Sparisoma cretense*, but its biomass was much lower than *Siganus*; and *Sarpa salpa*, which was not observed within our transects at the study sites. The carnivore sea bream *Diplodus sargus* can also consume moderate amounts of algae at our study habitat and depth (up to 10% of the weight of its stomach contents) [10]. However, this species does not reduce algal biomass dramatically elsewhere, even at the Medes Islands Marine Reserve, where its biomass is the largest in the Mediterranean, and more than 10 times larger than at our study sites [11].

These results suggest that an abundance of *Siganus* such as the observed (between 3 and 6 g m^−2^) may be sufficient to maintain benthic communities with extremely low algal biomass in the Mediterranean coast of Turkey. *Sarpa salpa* has been shown to maintain low algal cover in the NW Mediterranean [12], but not at the low levels encountered during this study.

**Figure 5:**
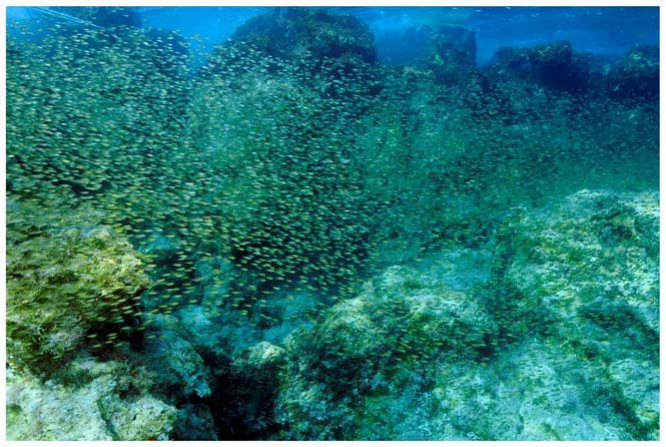
School of thousands of juvenile *Siganus luridus* at Kas, Turkey, after a massive recruitment event in September 2009.

**Figure 6:**
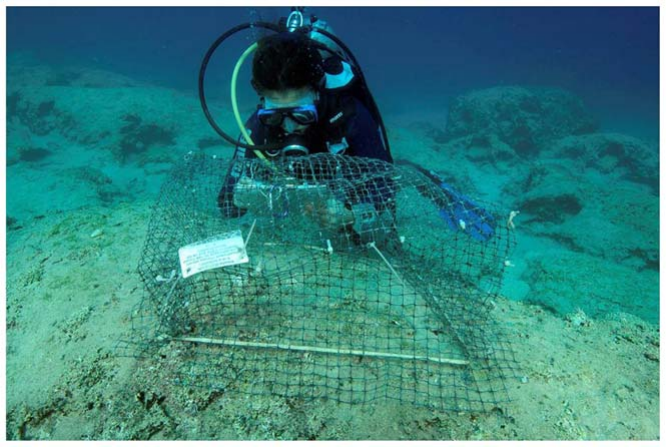
Experimental cages and surroundings, like this one at Kas, Turkey, were cleaned up by juvenile *Siganus* within a month.

Another question is whether *Siganus* can turn a well-developed algal assemblage into a barren, and how long it takes. We observed *Siganus rivulatus*, which recruited in massive numbers in September (Fig. 5), entering the cages and eating the algae, including large Fucales (Fig. 6), showing that *Siganus* have the potential to graze out well-developed algal canopies. Mediterranean algal assemblages exhibit seasonal changes in biomass, which typically declines after the ‘productive season’ (spring-summer) [13]. However, no infralittoral algal assemblage in the Mediterranean shows an abrupt decline to almost zero within a month [13] as we observed after *Siganus* grazing. Afterwards, the bottoms were devoid of visible algae and resembled a ‘true’ barren.

The grazing pressure needed to cause the shift from erect algae to ‘barren’ may be greater than that needed to maintain the barren. This difference has been well studied for the Mediterranean sea urchin *Paracentrotus lividus* [3] although we currently have no data for *Siganus*. Also, because of the absence of historical data on *Siganus* abundance and algal biomass on the Eastern Mediterranean, we cannot know when and why these barrens started to appear.

The two *Siganus* species consume the majority of macrophytes in the Eastern Mediterranean, although they prefer non-calcified algae and small filamentous algae, and have seasonal preferences [9]. However, they will eat whatever is available when algal biomass is lowest [14]. Why do *Siganus* stay on the barrens instead of moving to relatively close areas with larger algal biomass, and why they do not graze the uppermost sublittoral zone? Because *Siganus* have shown to be selective when macrophyte assemblages are diverse and abundant but they consume whatever is available in macroalgal-deprived bottoms [9], they may be maintaining microscopic carpets of filamentous algae and diatoms (‘gardening’ behavior).

Although we did not conduct a complete species list inside and outside the cages, we can assume that the loss of biodiversity due to fish grazing at the study sites is remarkable. Well-developed shallow water Mediterranean algal assemblages like those that grew inside the cages, dominated by a *Cystoseira* spp. canopy, can harbor up to 63–114 macroalgal species in an area of only 250–400 cm^2^ [3], [15]; whereas sea urchin barrens tend to have significantly fewer macroalgal species for the same area (as low as 23 species) [3].

The maximum biomass recorded in our cages was 625 g m^−2^ (wet mass) at Kas. Because that biomass arose from a baseline of less than 5 g m^−2^ in only 4 months, that maximum biomass is a conservative estimate of the potential annual primary production at Kas. These results suggest that the loss of primary production at the study habitat due to fish grazing may be considerable. However, we do not know the primary production provided by the small filamentous algae that *Siganus* appear to be cropping in the barrens (which can be relatively high in other habitats such as coral reefs). *Siganus* grazing should increase the turnover ratio in the depauperate barrens (i.e. the production/biomass ratio of the micro-turfs) but at the same time decrease total primary production. In any case, grazing causes a loss of biogenic structure that implies a loss of habitat for most marine invertebrates that live in algal canopies and that are the main prey items of the major carnivorous fishes on this habitat (Labridae, Sparidae and Serranidae) [10], [16], hence simplifying the food web. *Siganus* is directly transforming algae into faeces and shortcutting the complex Mediterranean food web at rocky infralittoral ecosystems [17], [18], [19].

The rapid growth of *Cystoseira* inside the cages was surprising because *Cystoseira* has relatively large zygots, and its propagule dispersal is thought to happen over very small distances (on the order of meters) [20]. First, there may have been small *Cystoseira* bases in small anfractuosities on the substrate, which had not been grazed by fish and could have grown the thalli we observed inside the cages. Second, propagules may have sunk from the wave-washed zone (upper infralittoral), where there was a narrow belt of *Cystoseira*. In any case, the rapid growth of *Cystoseira* suggests that recovery of algal assemblages is possible in the short term, should grazing pressure from *Siganus* be reduced.

Our results strongly suggest that grazing by the alien fish *Siganus luridus* and *S. rivulatus* creates and maintains areas denuded of erect algae in the Eastern Mediterranean, causing a dramatic reduction in biodiversity, biomass, and algal growth, with effects that may move up the food chain to the local fisheries. We hypothesize that a targeted experimental *Siganus* fishery in Turkey will help shift barrens to well-developed algal assemblages and enhance the recovery of the associated fauna. In the summer season, Lebanese fishermen collect the red alga *Palisada* sp. (quoted as *Chondrophycus papillosus)* (preferred by *Siganus* but largely unavailable to them in the field because the algae inhabit littoral Vermetid platforms) in large quantities and use it in traps as bait to capture specifically Siganidae [21]. Similar techniques could be used in Turkey to test whether the removal of the two alien marine fish leads to the restoration of the biogenic structure and biodiversity of Turkey's rocky coasts.

## Methods

### Study sites

The study was conducted at three sites on the Mediterranean coast of Turkey (Kas, Fethiye, and Bodrum) where we observed extensive barrens without sea urchins (Fig. 2). Sampling was conducted at an average depth of 10 m (±2 m) on rocky substrates with sub-horizontal slopes.

### Fish abundance

Fish data were collected by using standard underwater visual census at six stations at each site, separated at least 1 km apart from the next, in May 2009. We conducted 3 replicate 25 m-long and 5 m-wide transects at each station. Along each transect, the diver swam one way at constant speed, identifying and recording the number and size of each fish encountered [22]. Fish sizes were estimated visually in 5 cm increments of total length (TL). Fish biomass (wet weight) was estimated from size data by means of length-weight relationships from the available literature and existing databases [23].

For our analysis, we assigned each fish taxon to one of 4 trophic groups using the information about diet in the literature [23], and in previous Mediterranean studies [24]: apex predators, carnivores, herbivores, and (zoo)planktivores. To test for differences in biomass between trophic groups within each site, we performed a 2-way ANOVA, with site and station as random factors, and station nested within site.

### Fish exclusion experiment

To test whether the exclusion of fish grazing results in algal growth we conducted caging experiments at the three sites with extensive barrens that had no sea urchins (Kas, Fethiye, and Bodrum). We ran the experiments between March and November 2009 at Bodrum, but cages unexpectedly disappeared in August at Fethiye and in September at Kas. We installed twelve 60×40×25 cm plastic cages with mesh 2.5 cm in size. Potential caging artifacts include reduction of irradiance and water flow by the cages (which could inhibit algal growth inside the cages). Sala and Boudouresque experimentally found that this type of plastic cage does not significantly reduce irradiance of water flow, hence the absence of a control for caging artifacts in our study [11].

Once a month, we lifted the cages and photographed cages and an equal number of nearby control quadrats using a digital camera installed on a quadrapod. We measured the cover of algal taxa in the lab using Photogrid, and calculated algal biomass using the relationship between cover and biomass in the literature [13] and our own samples on nearby sites to account for the increase in biomass with height for the larger algae. The biomass of crustose coralline algae was not measured because they were not observable on the photographs when the canopy algae were present. In addition, we conducted sea urchin counts by placing fifty 50×50 cm quadrats haphazardly within 50 m of the enclosures at each site.

To test for differences in algal biomass between enclosures and control plots over time (between April and August) we performed a 3-way ANOVA, with time, treatment (cage vs. control), and site (Fethiye, Kas, Bodrum) as independent variables.
